# Airways Abnormalities in a Prospective Cohort of Patients With Rheumatoid Arthritis

**DOI:** 10.1016/j.chest.2024.09.006

**Published:** 2024-09-27

**Authors:** Scott M. Matson, Jiwoong Choi, Drayton Rorah, Shamir Khan, Anna Trofimoff, Taewon Kim, David H. Lee, Asma Abdolijomoor, Maggie Chen, Imaan Azeem, Linh Ngo, Tami J. Bang, Peter Sachs, Kevin D. Deane, M. Kristen Demoruelle, Mario Castro, Joyce S. Lee

**Affiliations:** a Department of Pulmonary, Critical Care and Sleep Medicine, University of Kansas Medical Cente, Kansas City, KS; b Department of Internal Medicine, University of Kansas Medical Center, Kansas City, KS; c University of Kansas School of Medicine, Kansas City, KS; d Department of Thoracic Radiology, National Jewish Hospital, Denver, CO; e Department of Radiology, University of Colorado Anschutz Medical Campus, Aurora, CO; f Division of Rheumatology, Department of Medicine, University of Colorado Anschutz Medical Campus, Aurora, CO; g Division of Pulmonary Sciences and Critical Care Medicine, Department of Medicine, University of Colorado Anschutz Medical Campus, Aurora, CO

**Keywords:** airways disease, asthma, COPD, quantitative CT imaging analysis, rheumatoid arthritis

## Abstract

**Background:**

Rheumatoid arthritis (RA) affects roughly 1% of the population and commonly involves the lungs. Of lung involvement in RA, interstitial lung disease (ILD) is well known; however, airways disease in RA is relatively understudied.

**Research Question:**

What are the baseline airways abnormalities in a prospective cohort of patients with RA based on pulmonary function testing (PFT) results, high-resolution CT (HRCT) scans, and computational imaging analysis and are there associations between these abnormalities and respiratory symptoms?

**Study Design and Methods:**

In this single-center study, 188 patients with RA without a clinical diagnosis of ILD underwent HRCT imaging and PFT. Radiologists assessed HRCT scans for airway abnormalities. Computational imaging via VIDA Vision software and in-house quantitative CT imaging analysis was applied to 147 HRCT scans to quantify airway abnormalities.

**Results:**

Airways obstruction (FEV_1_ to FVC ratio < 0.7) was present in 20.7% of patients and was associated with older age, male sex, and higher smoking rate. Radiologists identified airway abnormalities in 61% of patients: 55% had bronchial wall thickening, 12% had bronchiectasis, and 5% had mosaic attenuation. These airways findings were associated with older age; male sex; lower FEV_1_, FVC, and FEV_1_ to FVC ratio; and higher rates of rheumatoid factor positivity. Prespecified quantitative CT scan metrics (wall thickening percentage and emphysema percentage) correlated with obstruction in PFT results and more severe respiratory symptoms, including shortness of breath and cough.

**Interpretation:**

High rates of airways abnormalities were found in this prospective RA cohort based on three methods of detection. Significant associations were identified between quantitative CT scan measures and respiratory symptoms. Airways disease may be an underrecognized extra-articular manifestation of RA and quantitative CT imaging may be a sensitive method to detect the clinical impact on respiratory symptoms.

FOR EDITORIAL COMMENT, SEE PAGE 309

**Take-home Points**

**Study Question:** What are the baseline rates of airways abnormalities in a rheumatoid arthritis cohort and what are the clinical associations with respiratory symptoms?

**Results:** Patients with rheumatoid arthritis show high rates of airways abnormalities based on spirometry (20.7%) and radiologist-determined CT scan interpretation (61%), but only quantitative CT imaging analysis was associated with worse pulmonary symptoms.

**Interpretation:** Our results indicate that airways involvement in patients with rheumatoid arthritis is an underrecognized phenomena and has clinical importance based on associations with respiratory symptoms.

Rheumatoid arthritis (RA) is a common inflammatory arthritis affecting 1% of the general population.^1^ Although arthritis is the hallmark of RA, extra-articular manifestations of RA occur, and among these, lung disease is the leading cause of death in patients with RA.^2^, ^3^, ^4^, ^5^, ^6^ Despite the important clinical impact and high frequency of lung disease in patients with RA, little is known about airways disease in patients with RA compared with other pulmonary manifestations of RA, such as interstitial lung disease (ILD).

Patients with RA have higher incidence of asthma and COPD compared with control participants without RA matched for age and smoking history.^7^, ^8^, ^9^, ^10^, ^11^ Further, the presence of obstructive lung disease in patients with RA is associated with increased mortality.^12^, ^13^, ^14^ Unlike RA with ILD, RA airways disease (RA-AWD) typically is treated as an entity independent from the RA. That is, a patient with RA receives a diagnosis of COPD or asthma, as opposed to having an underlying manifestation of RA. It remains unknown if RA-AWD represents an aspect of disease that potentially has shared risk factors for articular diseases; for example, smoking is known to increase the risk of articular RA as well as to drive airways disease. Further, it is unknown if RA-AWD is a unique RA manifestation that further would respond to RA disease-modifying antirheumatic drugs, or if it should remain in the realm of typical pulmonologist-directed obstructive lung disease therapeutic approaches.

Using a prospective cohort of patients with RA,^15^ we sought to characterize airways abnormalities among patients with RA using standard physiologic and radiologic measures, as well as novel quantitative imaging analysis. Our hypothesis was that patients with RA exhibit findings of airways abnormalities and that the presence of airways abnormalities is associated with the presence and severity of respiratory symptoms.

## Study Design and Methods

Patients with RA were identified from a single-center outpatient rheumatology clinic at the University of Colorado Anschutz Medical Campus. Patients were asked to participate in the parent study^15^ if they had a diagnosis of RA based on the 2010 American College of Rheumatology criteria, a clinical diagnosis of RA by a board-certified rheumatology practitioner, or both. Patients were included in this cohort based on the absence of a clinical diagnosis of ILD. Patients with coexisting asthma, COPD, or bronchiectasis were not excluded from the parent study, and the study criteria did not exclude patients based on the presence or absence of respiratory symptoms. The institutional review board approved all protocols (Identifier: COMIRB 16-1907), and all patients provided written informed consent.

All patients filled out questionnaires focused on their medical history, smoking and exposure history, respiratory symptoms, and medications. Breathlessness was measured using the University of California, San Diego, Shortness of Breath Questionnaire (SOBQ).^16^ Cough severity was measured by the cough severity visual analog scale (VAS).^17^ RA disease activity was measured via Disease Activity Score—28 joint count C-reactive protein scores.^18^^,^^19^ Joint examinations were performed at the enrollment visit for all patients by board-certified rheumatologists (K. D. D., M. K. D.). Blood samples were collected from all patients, lung function measurements were obtained, and lung imaging was performed with high-resolution CT (HRCT) imaging.

Lung function was measured according to *European Respiratory Journal* and American Thoracic Society guidelines.^20^ Patients underwent spirometry (FEV_1_ and FVC) and diffusing capacity of the lungs for carbon monoxide % predicted maneuvers. Based on 2013 chronic obstructive lung disease guidelines, an FEV_1_ to FVC ratio of < 0.7 was used to determine the presence of airflow obstruction.^21^

All HRCT scans were read independently by two chest radiologists (T. J. B., P. S.) using a predetermined scoring form. Qualitative findings of airway abnormalities were scored and included assessment of bronchial wall thickening, emphysema, bronchiectasis, and mosaic attenuation abnormality. Discrepancies were resolved by consensus.

HRCT scans that met quality evaluations (147/183) underwent quantitative CT imaging analysis using Vision 2.2 (VIDA Diagnostics) and in-house quantitative CT imaging software for segmentation and measurements of airways, lungs, and lobes. Seventy-one airway and lung parenchymal structural features were computed for each patient, including percentage of emphysema, tissue fraction, percent of high attenuation area, airway wall thickness scaled as the inner perimeter of 10 mm, airway circularity, eccentricity, luminal area, wall area, wall area percent, wall thickness, wall thickness percentage, diameter, and so on.

We chose two of these quantitative imaging variables based on biologic rationale a priori to limit the impact of multiplicity in the subsequent analyses. The two variables for our targeted quantitative CT imaging analysis were wall thickening percentage and percentage of emphysema. Wall thickness percentage measures the percentage of all airway area in the total airway cross-sectional area (lumen plus wall). This measurement has been associated with important clinical outcomes and histologic findings of airway wall thickening in obstructive lung disease states.^22^^,^^23^ Percentage of emphysema is a quantitative measure of the percentage of lung volume of low attenuation (< –950 Hounsfield units) and has been associated with respiratory symptoms and worse pulmonary function measures in obstructive lung disease.^24^^,^^25^

Serum samples collected at the study visits were tested by enzyme-linked immunosorbent assay for rheumatoid factor (QUANTA Lite RF IgM; Inova Diagnostics, Inc.) and anticyclic citrullinated protein (cyclic citrullinated peptide 3.1 [IgA/G]; Inova Diagnostics, Inc.) with cutoffs for positivity for each test determined according to the manufacturer’s specifications. High-sensitivity C-reactive protein testing was performed using nephelometry (DadeBehring).

### Statistical Analysis

Baseline demographics were described using median (interquartile range) and proportions, as appropriate. Comparisons between groups were carried out using χ^2^ tests or Mann-Whitney *U* tests, as appropriate based the evaluation of continuous or categorical variables. Significance of overlap of patients with spirometry-determined obstruction and radiologist-defined airways abnormalities were carried out using the Fisher exact test. Using the SOBQ scores and the cough severity VAS system^16^^,^^17^^,^^26^^,^^27^ as independent outcome measures, we developed univariate and multivariate linear regression models to assess the association with the various measures of airways abnormalities (spirometry and qualitative and quantitative radiologic variables) as exposure variables, adjusting (in a single multivariate model) for age, male sex, history of ever smoking, and history of previously diagnosed obstructive lung disease. Univariate associations between quantitative CT imaging measures and clinical variables, as well as quantitative CT imaging measures and radiologist determinations and spirometry obstruction, were determined by Pearson’s product-moment correlation test for continuous-continuous associations and point-biserial correlation coefficients for binary-continuous associations. All statistical analysis was performed in R version 091+494 software (R Foundation for Statistical Computing) with a significance threshold of *P* < .05.

## Results

The characteristics of the 188 patients included in the study are reported in Table 1.^16^^,^^18^ Most patients were female, were non-Hispanic White, and had active or former tobacco use (Table 1). Most patients showed seropositive results for rheumatoid factor and anticyclic citrullinated protein. Patients who previously received a diagnosis of asthma or COPD constituted 19.6% and 5.3% of the study patients, respectively. The median time between a diagnosis of RA and the study was 8.5 years, and most patients were taking methotrexate at the time of study enrollment. Although 188 total patients contributed data to these analyses, five patients did not undergo spirometry and a different five patients elected not to undergo CT imaging. Therefore, all subsequent analyses were based 183 patients.

**Table 1:** Baseline Characteristics

Variable	Data
Total No. of patients	188
Age, y^a^	53.8 (14.9)
Male sex	36 (19%)
Non-Hispanic White	137 (73%)
Smoking history	
Current	34 (19%)
Never	98 (52%)
Ever	90 (48%)
Pack-y among those who ever smoked	10.8 (22.3)
Diagnosis of asthma	37 (20%)
Diagnosis of COPD	10 (5%)
FEV_1_, % predicted	98 (23)
FVC, % predicted	100 (42)
FEV_1_ to FVC ratio	77 (22)
Dlco, % predicted	87 (34)
Rheumatoid factor positive	152 (81%)
Anticyclic citrullinated peptide antibody positive	161 (86%)
UCSD SOBQ score^b^	6 (16)
DAS-28 CRP^c^	2.7 (1.8)
RA duration, y	8.5 (14)
RA treatment (current at enrollment)	
Methotrexate	98 (52%)
Other conventional DMARD^d^	83 (44%)
Biologic DMARD^e^	96 (51%)
Prednisone	123 (66.8%)

Data are presented as No. (%), mean (SD), or median (interquartile range). DAS-28 CRP = Disease Activity Score with 28 joint count and C-reactive protein; Dlco = diffusing capacity of the lungs for carbon monoxide; DMARD = disease-modifying antirheumatic drug; RA = rheumatoid arthritis; SOBQ = Shortness of Breath Questionnaire; UCSD = University of California, San Diego.

a Based on measures of skewness.

b Scale of 0-120, with higher scores indicating more breathlessness.^16^

c Scale of 2-10, with scores of < 2.6 representing remission and scores of > 5.1 indicating high disease activity.^18^

d Other conventional DMARDs (other than methotrexate) include: leflunomide, sulfasalazine, hydroxychloroquine, azathioprine, and mycophenolate mofetil.

e Biologic DMARDs include: etanercept, adalimumab, certolizumab, golimumab, infliximab, rituximab, abatacept, and tocilizumab.

### Assessment of Airways Abnormalities Across the Cohort

#### Physiologic Assessment of Airways Abnormalities

Thirty-eight of 183 patients (20.7%) with RA showed an FVC to FEV_1_ ratio of < 0.7 based on consensus obstructive lung disease guidelines (Table 2).^21^ When removing the patients with RA who reported previously receiving a diagnosis of asthma (n = 37) or COPD (n = 10; including three patients with a diagnosis of overlap asthma and COPD), we observed that 26 of the remaining 146 patients (18.9%) showed spirometry-based airflow obstruction.

**Table 2:** Spirometry Features of Cohort Based on Presence of Obstruction (FEV_1_ to FVC Ratio < 0.7)

Variable	Obstructed (n = 38; 20.7%)	Nonobstructed (n = 145; 79.3%)	*P* Value
Age, y	61.6 (12.7)	50.0 (19.2)	***<* .01**^a^
Male sex	11 (29%)	20 (14%)	.06^b^
History of ever smoking	30 (81%)	60 (42%)	***<* .01**^b^
FEV_1_, % predicted	76.5 (62.6, 90.4)	100 (90, 110)	***<* .01**^a^
FVC, % predicted	95 (82.5, 107.5)	101 (90.5, 111.5)	**.03**^a^
Dlco, % predicted	79 (64, 94)	89 (76.35, 101.65)	***< .*01**^a^
Rheumatoid factor positive^c^	34 (89%)	112 (78%)	.11^b^
Anticyclic citrullinated peptide antibody positive (cyclic citrullinated peptide 3.1)^c^	30 (79%)	122 (84%)	.6^b^
Current methotrexate use	23 (61%)	71 (49%)	.3^b^
RA mean duration, y	13.6 (11.3)	12.7 (11.7)	.6^a^
DAS-28 CRP^d^	2.7 (2.0)	2.6 (1.6)	.9^a^

Data are presented as No. (%), mean (SD), or median (quartile 1, quartile 3) unless otherwise indicated. Bold indicates significant *P* value (*P* < .05). DAS-28 CRP = Disease Activity Score with 28 joint count and C-reactive protein; Dlco = diffusing capacity of the lungs for carbon monoxide; RA = rheumatoid arthritis.

a Mann-Whitney *U* test.

b Pearson χ^2^ test for proportion comparisons.

c Missing in six individuals because of lack of consent for research blood draws (ie, missing at random).

d Missing in 36 individuals.

Patients with obstruction were significantly older, had significantly greater rates of having ever used tobacco, and had significantly lower FEV_1_ % predicted, FVC % predicted, and diffusing capacity of the lungs for carbon monoxide % predicted results compared with those without obstruction. No significant differences were found in seropositivity, current methotrexate use, or RA disease activity or duration across the two groups.

We then evaluated the relationship between the presence of obstruction on spirometry and respiratory symptoms as measured by the SOBQ and cough severity VAS scores. We found no significant association between obstruction pattern on spirometry and shortness of breath or cough severity when adjusting for age, smoking history, sex, and history of prior obstructive lung disease diagnosis (Table 3).

**Table 3:** Univariate and Multivariate Association Between Shortness of Breath and Cough Severity With Spirometry Obstruction, Radiologist-Determined Airways Abnormalities, Wall Thickness, and Emphysema Percent

Method	UCSD SOBQ	Cough Severity VAS
Spirometry		
Univariate association	β = 3.6, *P* = .3	β = 39.6, *P* = .6
Multivariate association	β = 3.7, *P* = .3	β = 65.3, *P* = .4
Radiologist-defined abnormalities		
Univariate association	β = –4.3, *P* = .1	β = –4.3, *P* = .4
Multivariate association	β = –3.4, *P* = .2	β = –4.9, *P* = .3
Wall thickness percentage		
Univariate association	**β = 2.2, *P* < .01**	β = 2.0, *P* = .09
Multivariate association	**β = 1.8, *P* < .01**	β = 2.0, *P* = .06
Percentage of emphysema		
Univariate association	**β = 1.3, *P* < .01**	**β = 1.1*****P*** **= .01**
Multivariate association	**β = 1.3, *P* = .01**	**β = 1.3, *P* = .01**

Bold indicates significant *P* value (*P* < .05). SOBQ = Shortness of Breath Questionnaire; UCSD = University of California, San Diego; VAS = visual analog scale.

#### Radiologist Assessment of Airways Abnormalities

**Figure 1:**
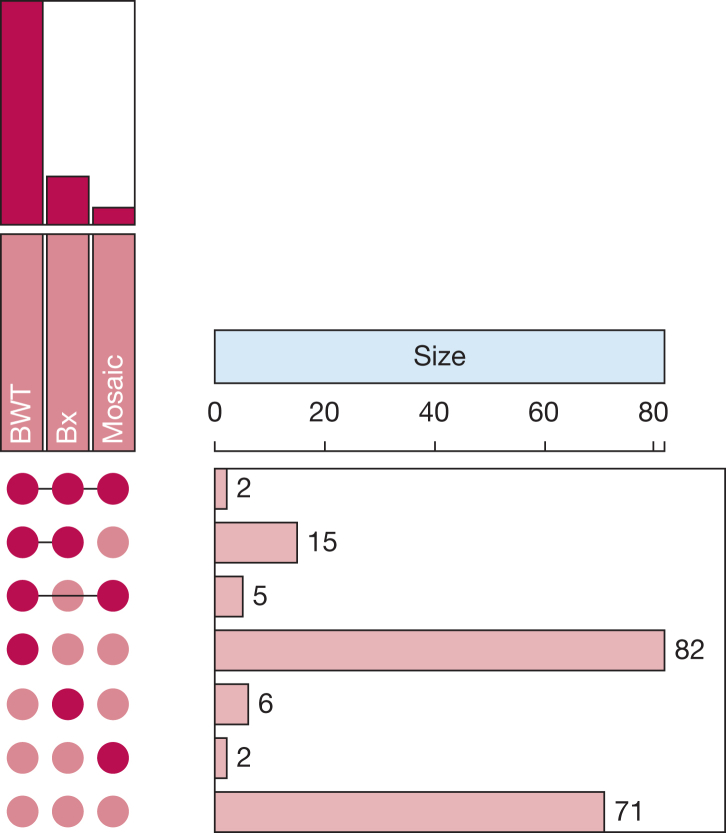
Upset plot showing the three categories listed vertically: BWT, Bx, and mosaic attenuation abnormality. Each set is represented by a circle or dot. If a category is present in a combination, its corresponding circle is filled; if it is absent, the circle is empty. The horizontal bars represent the size of the intersection between the sets. The connected dots above each bar indicate which sets are being intersected. For example, a bar with a connected dot under Bx, but not under BWT or Mosaic, represents the number of items that are only in the Bx set. The length of the bar corresponds to the number of items in the intersection and the numbers on the right indicate the exact size (count) of the intersection they are aligned with. For instance, referring to the plot, the bar connected with filled circles under Bx and Mosaic, but not under BWT, indicating that the number of items that are in both BWT and Mosaic sets, but not in Bx, is 15, indicates that 15 patients in this cohort had both BWT and mosaicism, but no bronchiectasis. BWT = bronchial wall thickening; Bx = bronchiectasis.

**Figure 2:**
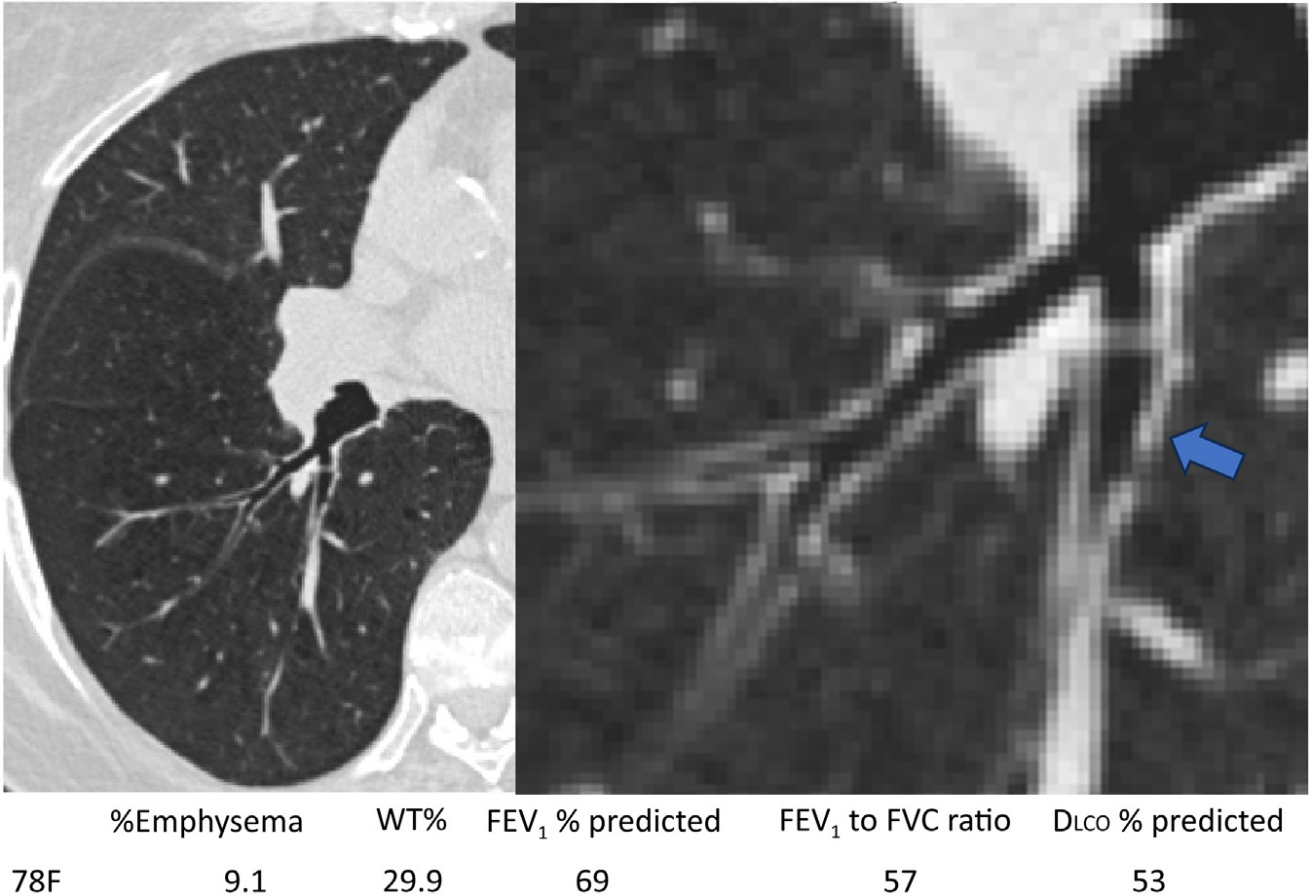
CT scans of airways of representative patients with bronchial wall thickening denoted by blue arrow. Below the figure, quantitative CT imaging variables also are included (%Emphysema and WT%), in addition to pulmonary function testing values (FEV_1_, FEV_1_ to FVC ratio, and Dlco % predicted). Dlco = diffusing capacity of the lungs for carbon monoxide; %Emphysema = percentage of emphysema; WT% = wall thickness percentage.

At least one consensus airways abnormality was identified on HRCT imaging in 112 of 183 patients (61%). Among these 112 patients, bronchial wall thickening was present in 104 of 183 patients (57%), 23 patients (13%) showed bronchiectasis without associated traction, and nine patients (5%) showed mosaic attenuation (Fig 1); 22 patients showed > 1 airway finding. A representative image is included for an example of each of these findings in Figure 2. Patients with radiologist-defined airways abnormalities were significantly older, a significantly higher proportion were male, and this group showed significantly lower FEV_1_, FVC, and FEV_1_ to FVC ratio findings, in addition to a significantly greater proportion showed positive rheumatoid factor results (Table 4).

**Table 4:** Radiologist-Defined Airways Abnormalities

Variable	Radiologist-Determined Airway Abnormality (n = 112)	No Radiologist-Determined HRCT Imaging Airways Abnormalities (n = 71)	*P* Value
Age, y	55.6 (15)	50.63 (14.3)	**.03**^a^
Male sex	25 (22.5%)	7 (9.9%)	***<* .01**^b^
History of ever smoking	60 (57.6%)	27 (40.9%)	.2^b^
FEV_1_, % predicted	95 (82.5, 107.5)	100 (88.8, 111.3)	***<* .01**^a^
FVC, % predicted	99 (89.8, 108.3)	103 (91.1, 114.9)	**.03**^a^
Dlco, % predicted	85 (71.8, 98.3)	90 (77.6, 102.4)	.2^a^
FEV_1_ to FVC ratio	0.75 (0.1)	0.8 (0.09)	***<* .01**^a^
Rheumatoid factor positive	96 (85.7%)	50 (70.4%)	**.02**^b^
Anticyclic citrullinated peptide antibody positive	95 (84.8%)	57 (80.2%)	.6^b^
Current methotrexate use	63 (58%)	32 (45.7%)	.1^b^
RA duration, y	10.5 (14.75)	9 (14.5)	.4^a^
DAS-28 CRP	2.7 (2.1)	2.5 (1.4)	.5^a^

Data are presented as No. (%), mean (SD), or median (quartile 1, quartile 3) unless otherwise indicated. Airways abnormalities identified were not mutually exclusive (bronchial wall thickening, n = 105; bronchiectasis, n = 23; mosaic attenuation, n = 9). Bold indicates significant *P* value (*P* < .05). DAS-28 CRP = Disease Activity Score with 28 joint count and C-reactive protein; Dlco = diffusing capacity of the lungs for carbon monoxide; HRCT = high-resolution CT; RA = rheumatoid arthritis.

a Mann-Whitney *U* test.

b Pearson χ^2^ test for proportion comparisons.

Next, we evaluated the association between the radiologist-defined airways abnormalities and the respiratory symptoms shortness of breath and cough severity using the SOBQ and cough severity using the cough severity VAS (Table 3). Further, we explored individual components of the HRCT imaging airways features for these associations in a sensitivity analysis (e-Table 1). In both the unadjusted and adjusted models, we found no significant association between HRCT imaging-determined airways abnormalities and shortness of breath or cough severity. Only mosaic attenuation was associated independently with shortness of breath (SOBQ: β = 17.74; *P* < .01) (e-Table 1).

#### Quantitative Imaging Assessment of Airways Abnormalities

**Figure 3:**
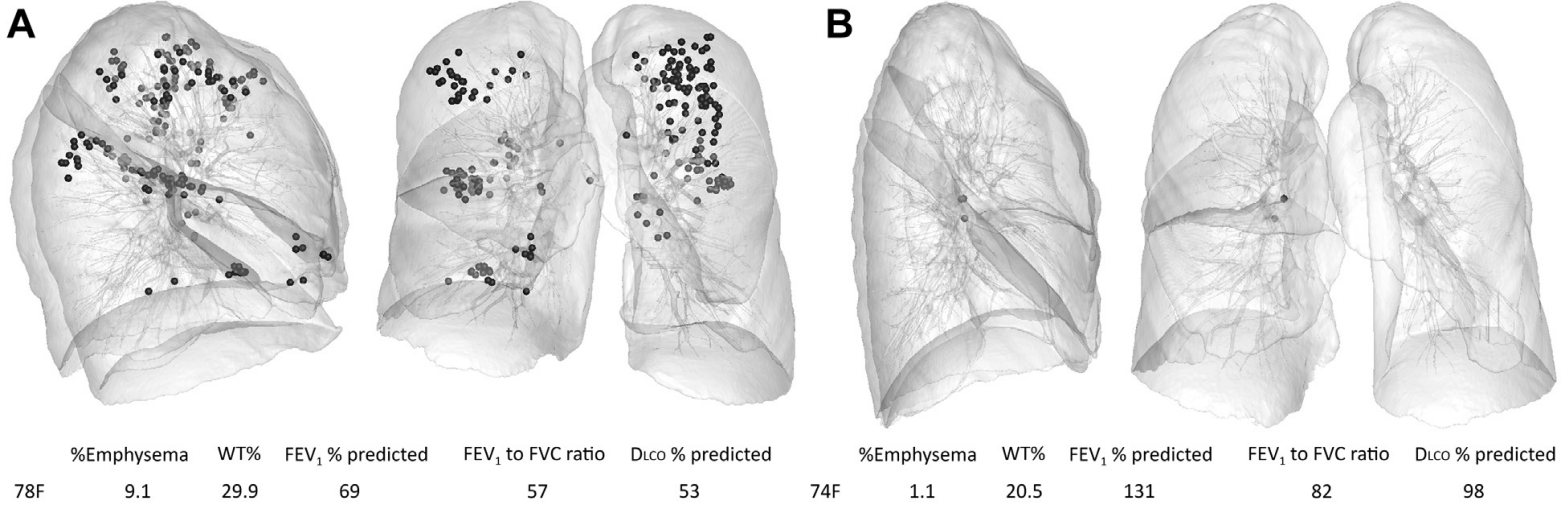
Images of representative patients with high and low scores of WT% and %Emphysema: dark spheres indicate quantitative CT imaging-based emphysematous regions. Dlco = diffusing capacity of the lungs for carbon monoxide; %Emphysema = percentage of emphysema; WT% = wall thickness percentage.

Of the 183 patients with HRCT imaging available, 147 patients (80.3%) had HRCT scans that met technical criteria to undergo quantitative CT imaging analysis. The median wall thickness percentage was 25.3 (IQR, 3.43 [23.4-26.8]) and the median percentage of emphysema was 1.4 (IQR, 2.8 [0.7-3.5]). Representative images for wall thickness percentage and percentage of emphysema are presented in Figure 3.

In univariate analyses, a significant association was found between wall thickness percentage and lower FEV_1_ % predicted as well as longer RA duration and higher RA disease activity. Percentage of emphysema was associated significantly with older age, lower diffusing capacity of the lungs for carbon monoxide % predicted, lower FEV_1_ to FVC ratio, and higher RA disease activity (Table 5).

**Table 5:** Univariate Association of Wall Thickness Percentage and Percentage of Emphysema With Clinical Variables

Variable	Univariate Association With Wall Thickness Percentage	Univariate Association With Percentage of Emphysema
Age	–0.1 (–0.3 to 0.04) *P* = .1	**0.3 (0.1–0.4) *P* < .01**
Male sex	–0.1 (–0.3 to 0.02) *P* = .09	–0.1 (-0.3 to 0.06) *P* = .2
History of ever smoking	0.09 (–0.07 to 0.3) *P* = .3	0.2 (-0.02 to 0.3) *P* = .07
FEV_1_, % predicted	**–0.2 (–0.3 to –0.2) *P* = .03**	–0.1 (-0.3 to 0.05) *P* = .3
FVC, % predicted	0.09 (–0.3 to 0.07) *P* = .3	0.01 (-0.2 to 0.2) p =0.9
Dlco, % predicted	0.004 (–0.2 to 0.15) p=1.0	**–0.2 (–0.4 to –0.06) *P* < .01**
FEV_1_ to FVC ratio	–0.2 (–0.3 to 0.00) *P* = .05	**–0.3 (–0.5 to –0.2) *P* < .01**
Rheumatoid factor positive	0.08 (–0.08 to 0.2) *P* = .3	0.06 (–0.1 to 0.2) *P* = .5
Anticyclic citrullinated peptide antibody positive	0.1 (–0.04 to 0.3) *P* = .1	–0.02 (–0.2 to 0.2) *P* = .8
Current methotrexate use	0.11 (–0.05 to 0.3) *P* = .2	0.05 (–0.1 to 0.2) *P* = .5
RA duration, y	**0.2 (0.05–0.4) *P* = .01**	–0.05 (–0.2 to 0.1) *P* = .5
DAS-28 CRP	**0.2 (0.02–0.4) *P* = .03**	**0.2 (0.03–0.4) *P* = .02**

Univariate associations between quantitative CT imaging measures and clinical variables, as well as quantitative CT imaging measures and radiologist determinations and spirometry obstruction were determined by Pearson product-moment correlation test for continuous-continuous associations and point-biserial correlation coefficients for binary-continuous associations. Bold indicates significant data presented as effect size (confidence interval) *P* value. DAS-28 CRP = Disease Activity Score with 28 joint count and C-reactive protein; Dlco = diffusing capacity of the lungs for carbon monoxide; RA = rheumatoid arthritis.

In univariate analysis, we found a significant positive association between wall thickness percentage and SOBQ scores (β = 2.2; *P* < .01). Using a multivariate model, this association remained significant after adjustment for age, sex, smoking history, and previous diagnosis of COPD or asthma (β = 1.79; *P* < .01). A trend for higher wall thickness percentage was found in those with worse cough severity scores, although this did not meet statistical significance (univariate: β = 2.0; *P* = .087; multivariate: β = 2.0; *P* = .06).

Percentage of emphysema also was associated significantly with SOBQ scores in the univariate and multivariate analyses (univariate: β = 1.3; *P* < .01; multivariate: β = 1.3; *P* = .01) and worse cough severity scores (univariate: β = 1.1; *P* = .01; multivariate: β = 1.3; *P* = .01).

To assess this association further, we performed a sensitivity analysis using only those who had no history of smoking in the RA cohort (n = 75 [51%]). In those with RA who had never smoked, we identified a significant univariate association between both wall thickness percentage and percentage of emphysema with SOBQ scores (univariate wall thickness percentage: β = 2.0; *P* = .03; univariate percentage of emphysema: β = 1.4; *P* = .04). In multivariate analyses, both associations remained significant (wall thickness percentage: β = 1.8; *P* < .01; percentage of emphysema: β = 1.4; *P* < .01). No significant univariate or multivariate associations were found between quantitative CT imaging metrics and cough severity VAS scores for those with RA who had no history of smoking.

### Comparison of PFT, HRCT Imaging, and Quantitative CT Imaging Findings

**Figure 4:**
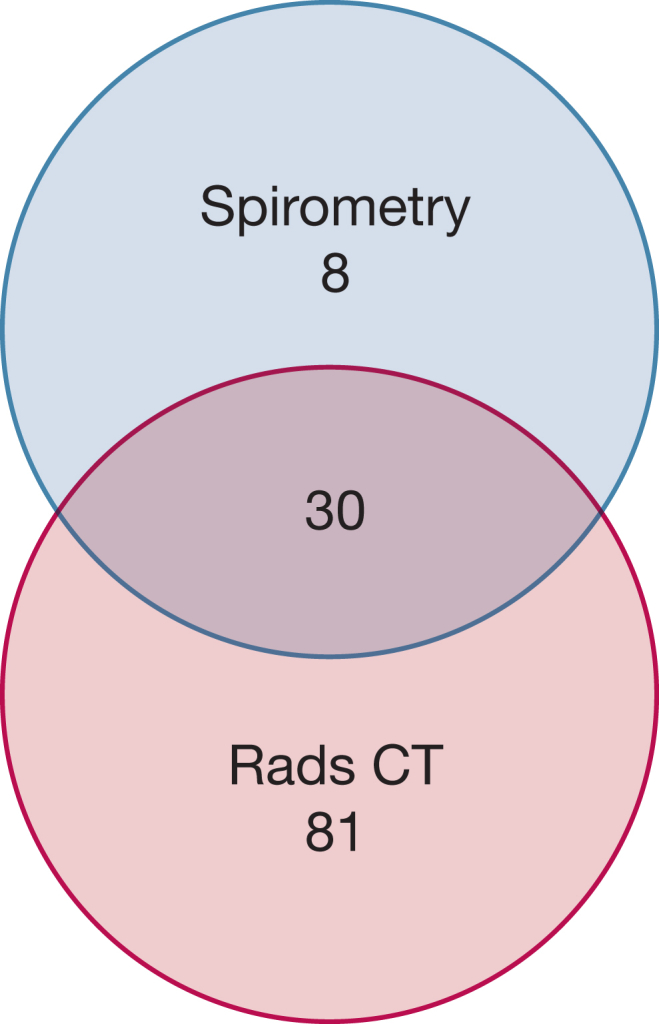
Venn diagram visualizing the relationship between patients who had spirometry obstruction without Rads CT (n = 8), patients with both features (n = 30), and patients with Rads CT, but no obstruction identified on spirometry (n = 81). Rads CT = radiologist-identified airways abnormalities.

A significant association was found between FEV_1_ to FVC ratio of < 0.7 and radiologist-determined HRCT imaging airways abnormalities (OR, 2.9; 95% CI, 1.17-7.72; *P* = .014). Thirty patients showed both obstruction on PFT and radiologist-determined airways abnormalities, whereas eight patients showed obstruction by spirometry with normal HRCT imaging findings and 81 patients showed airways abnormalities on HRCT imaging without obstruction on PFT results (Fig 4).

**Figure 5:**
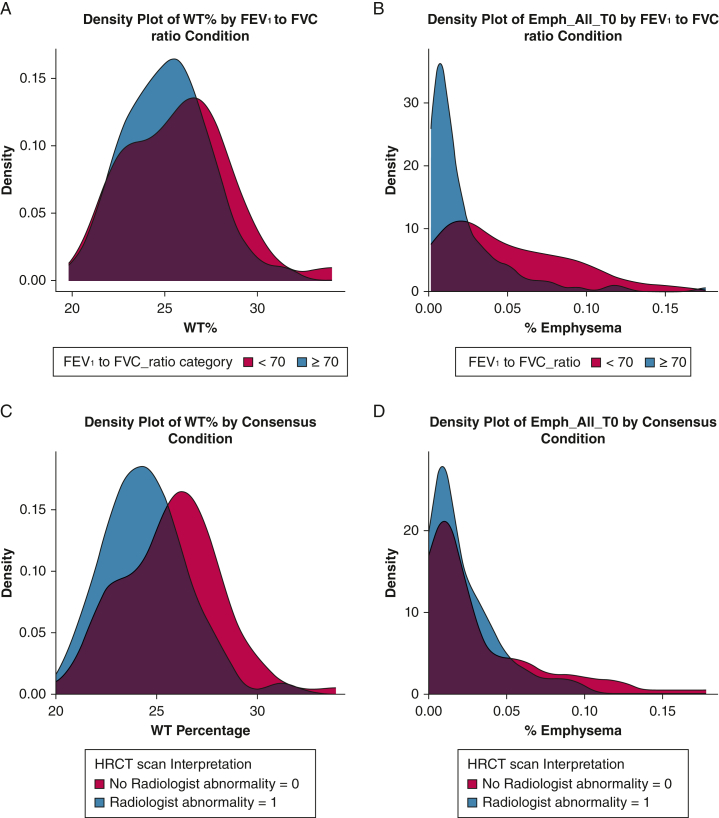
A-D, Density plots illustrating patient distributions. These graphs present kernel density estimates, which depict the probability density of patients’ quantitative CT imaging measurements. The x-axis represents the measured variable, either WT% or %Emphysema. The y-axis shows the estimated density of patients at each point along the variable scale. These plots allow for the visual comparison of distributions between the two distinct patient categories within the dataset. A, Visualizes the overlap between wall thickness percentage and spirometry where patients with obstruction are coded in red and patients without obstruction are coded in blue. B, Visualizes the overlap between percentage of emphysema and spirometry where patients with obstruction are coded in red and patients without obstruction are coded in blue. C, Visualizes the overlap between wall thickness percentage and radiologist consensus of imaging airway abnormality where patients with HRCT imaging abnormalities are coded in red and those with normal HRCT imaging findings are coded in blue. D, Visualizes the overlap between percentage of emphysema and radiologist consensus of imaging airways abnormality where patients with HRCT imaging abnormalities are coded in red and those with normal HRCT imaging findings are coded in blue. Emph_All_T0 = % emphysema (in whole lung); HRCT = high-resolution CT; %Emphysema = percentage of emphysema; WT% = wall thickness percentage.

A significant correlation was found between wall thickness percentage and spirometry-determined obstruction (*r* = –0.16; 95% CI, –0.31 to –0.01; *P* = .042) and a significant correlation was found between percentage of emphysema and obstruction by spirometry (β = –0.33; 95% CI, –0.47 to –0.18; *P* < .01; Fig 5). Wall thickness percentage was correlated significantly with radiologist-determined airways abnormalities (*r* = 0.25; 95% CI, 0.1–0.4; *P* < .01); however, no significant correlation was found between percentage of emphysema and radiologist-determined airways abnormalities (β = 0.13; 95% CI, –0.03 to 0.28; *P* = .12) (Fig 5).

## Discussion

In this prospective cohort, we identified airways abnormalities in patients with RA using three different methods of assessment: pulmonary physiologic features, radiologist interpretation of HRCT imaging, and quantitative CT imaging. In particular, 22% of all patients with RA (19% when removing patients with RA with a previous diagnosis of asthma or COPD) had obstructed pulmonary physiologic findings. Further, consensus qualitative radiologist interpretation of HRCT scans found high rates of radiologic airways abnormalities in patients with RA (61%). Both PFT findings and radiologist-determined airways abnormalities were not associated with symptoms of shortness of breath or cough severity.

Quantitative imaging (wall thickness percentage and percentage of emphysema) features were associated with respiratory symptoms in the analysis. If symptoms were used as a clinically meaningful outcome in the definition of RA-AWD, quantitative imaging increased sensitivity and specificity for airways disease when compared with a PFT definition of obstruction of FEV_1_ to FVC ratio of < 0.7 and radiologist interpretation of HRCT imaging. This difference suggests that although spirometry and radiologist interpretation of HRCT imaging are tools for detecting airways abnormalities, they may not fully capture more subtle changes that have functional impact on patients. Additionally, FEV_1_ to FVC ratio of < 0.7 primarily detects obstruction and air trapping; RA seems to have a more diverse impact on airways aside from obstruction alone, and different cutoffs of this ratio or more sensitive PFT measurements might detect higher rates of abnormalities. Given this, the significant association between wall thickness percentage and percentage of emphysema with respiratory symptom severity suggests that quantitative CT imaging could serve as a noninvasive biomarker for early clinically significant airway disease in RA, which could be leveraged for future prospective, interventional studies of RA airways disease. The future research agenda in RA-AWD should aim to develop consensus definitions and to validate methods of diagnosis such as quantitative CT imaging in large, longitudinal studies with an emphasis on identifying and predicting clinical outcomes and therapeutic response.

Our findings are similar to previous descriptions of RA-AWD from a smaller prospective cohort of patients with RA who underwent spirometry and HRCT imaging that also found high rates of HRCT imaging measures of airways abnormalities in 24 of 50 patients^28^ and a second prospective analysis that found preclinical radiologic emphysema in 36% of 106 patients with RA.^29^ Our analysis builds on these findings by increasing the population studied, in addition to leveraging quantitative imaging features from HRCT imaging in a cohort with prospectively collected measures of respiratory symptoms to identify important clinical associations. Recently, investigators in Brazil published reports of longitudinal PFT trends for patients with RA-AWD identified from a large retrospective population with RA identified by HRCT imaging features of airways disease.^30^ These findings describe longitudinal increases in air trapping on serial spirometry measurements, further supporting the clinical importance of increased recognition of airways disease in patients with RA.

Further, the association between RA disease activity and quantitative measures of airways disease is intriguing. Compelling data point to the lung and airway mucosa as the site of origin of RA autoantibody production and RA risk based on chest imaging findings and sputum levels of RA autoantibodies that predict progression to RA in individuals with pre-RA and epidemiologic studies that highlight a bidirectional relationship between RA and airways disease.^31^, ^32^, ^33^, ^34^, ^35^ Interestingly, we confirmed a significant association between respiratory symptoms (SOBQ scores) and quantitative CT imaging metrics in patients with RA who had never smoked. This intriguing sensitivity analysis indicates that airways abnormalities and respiratory symptoms occur in patients with RA independent of the shared risk factor of smoking. In total, these data suggest that RA-AWD may represent an underrecognized pulmonary manifestation of RA, and therefore may respond to a disease-modifying antirheumatic drug approach. This is in contrast to the current standard of care, which typically is inhaled bronchodilator therapy or corticosteroid-based therapy (or no treatment at all). Longitudinal studies that target RA-AWD are necessary to understand the role of treatment in this group, and quantitative CT imaging may be an important method to detect and monitor these patients over time.

Our study has important limitations. First, our data describe rates of RA-AWD in a prospectively collected group of patients without ILD. It is possible that patients with RA and ILD also have high rates of airways involvement, given the high rates of airways involvement in other populations with ILD.^36^^,^^37^ Therefore, exclusion of patients with RA and ILD may not fully capture the extent and clinical impact of RA-AWD in an unbiased population with RA. Similarly, we included patients with RA with previous diagnoses of asthma and COPD in the primary cohort. The inclusion of these patients more accurately reflects the cross-sectional prevalence of RA-AWD in this prospective cohort (including those with previously diagnosed clinical asthma or COPD). Our hypothesis was that RA would be associated with airways disease as a primary inflammatory manifestation, and therefore, previously diagnosed asthma or COPD in an RA patient could represent an unrecognized RA manifestation. However, we used multivariate modeling that adjusted for a prior obstructive lung disease diagnosis.

## Interpretation

These data highlight the importance of airways involvement in patients with RA. Airways disease is common in RA, and quantitative measures of airways abnormalities are associated with clinically important symptoms of shortness of breath and cough severity, as well as RA disease activity. Our data support future study of this phenotype and longitudinal studies to understand the impact of treatment directed at this phenotype on patient symptoms and RA disease activity.

## Funding/Support

This work was funded by the 10.13039/100000057National Institute of General Medical Sciences, National Institutes of Health [Grant P20GM130423 to S. M. M.], the 10.13039/100000050National Heart, Lung, and Blood Institute, National Institutes of Health [Grant HL138131 to J. S. L.], and an investigator-initiated Pfizer Aspire Grant [Grant WI214990 to K. D. D.].

## Financial/Nonfinancial Disclosures

The authors have reported to *CHEST* the following: M. K. D. and K. D. D. report investigator-initiated grants from Pfizer. M. Castro accepts consulting fees from Genentech, Teva, Sanofi-Aventis, Merck, Novartis, Arrowhead Pharmaceuticals, Allakos, Amgen, OM Pharma, Pfizer, Pioneering Medicines, and GSK and honoraria from Amgen, AstraZeneca, Genentech, Regeneron, Sanofi-Aventis, and Teva. J. S. L. receives consulting fees from Galapagos, Boehringer Ingelheim, United Therapeutics, Eleven P15, and Bonac. None declare (S. M. M., J. C., D. R., S. K., A. T., T. K., D. H. L., A. A., M. Chen, I. A., L. N., T. J. B., P. S.).

## Supplementary Data

e-Online Data
