# Supplementary material for: Airways Abnormalities in a Prospective Cohort of Patients With Rheumatoid Arthritis
Source: Chest. 2024 Sep 27;167(2):495–506. doi: 10.1016/j.chest.2024.09.006 (PMC11867896; doi:10.1016/j.chest.2024.09.006)
Supplement: e-Online Data [file mmc1.docx]

| Bronchial Wall Thickening | Univariate | Multivariate |
| --- | --- | --- |
| UCSDSOB | β = 3.94, p =0.13 | β = 4.1, p =0.12 |
| VAS | β = 5.9, p =0.19 | β = 6.8, p =0.16 |
| Presence of cough | **β = 0.68, p =0.036** | **β = 0.72, p =0.031** |
| Bronchiectasis |  |  |
| UCSDSOB | β = 2.88, p =0.45 | β = 4.69, p =0.24 |
| VAS | β = 8.4, p =0.17 | β = 9.7, p =0.14 |
| Presence of cough | β = 0.14, p =0.76 | β = -0.12, p =0.81 |
| Mosaic Attenuation |  |  |
| UCSDSOB | **β = 19.4, p <0.01** | **β = 17.74 p <0.01** |
| VAS | β = -1.9, p =0.82 | β = -5.3, p =0.56 |
| Presence of cough | β = 0.67, p =0.36 | β = 0.14, p =0.76 |

E-Table 1
